# Ultrasound-Guided Versus Landmark-Based Extracorporeal Shock Wave Therapy for Calcific Shoulder Tendinopathy: An Interventional Clinical Trial

**DOI:** 10.3390/diagnostics15091142

**Published:** 2025-04-30

**Authors:** Iosif Ilia, Caius Calin Miuta, Gyongyi Osser, Brigitte Osser, Csongor Toth, Manuela Simona Pop, Ramona Nicoleta Suciu, Veronica Huplea, Victor Niculescu, Laura Ioana Bondar

**Affiliations:** 1 Department of Kinesitherapy and Special Motricity, Faculty of Physical Education and Sport, “Aurel Vlaicu” University of Arad, 310130 Arad, Romania; iosif.ilia@uav.ro (I.I.); caius.miuta@uav.ro (C.C.M.); brigitte.osser@uav.ro (B.O.); csongor.toth@uav.ro (C.T.); 2 Doctoral School of Biomedical Sciences, University of Oradea, 410087 Oradea, Romania; bondar.lauraioana@student.uoradea.ro; 3 Department of Psycho Neuroscience and Recovery, Faculty of Medicine and Pharmacy, University of Oradea, 410087 Oradea, Romania; manuela.pop@didactic.uoradea.ro (M.S.P.); ramona_suciu@uoradea.ro (R.N.S.); hupleaveronica@uoradea.ro (V.H.); 4 Doctoral School, University of Medicine and Pharmacy “Carol Davila” Bucuresti, 050074 Bucharest, Romania; victor.niculescu@drd.umfcd.ro; 5 Department of Biology and Life Sciences, “Vasile Goldiș” Western University of Arad, 310048 Arad, Romania

**Keywords:** calcific tendinopathy, Constant–Murley score, extracorporeal shockwave therapy, supraspinatus tendon, ultrasound-guided therapy

## Abstract

**Background/Objectives**: Calcific tendinopathy of the shoulder is a degenerative condition characterized by calcium deposits within the rotator cuff tendons, particularly the supraspinatus. It is a frequent cause of chronic shoulder pain and functional limitation, adversely affecting quality of life. While conservative treatments such as nonsteroidal anti-inflammatory drugs (NSAIDs), physiotherapy, and corticosteroid injections are commonly used, extracorporeal shock wave therapy (ESWT) has emerged as a promising non-invasive alternative. This interventional clinical trial compared the efficacy of ultrasound-guided versus landmark-based ESWT in treating calcific tendinopathy. **Methods**: Eighty-four patients with ultrasound-confirmed calcific tendinopathy were randomized into two groups. Group 1 received ultrasound-guided ESWT with real-time targeting of the deposit; Group 2 received landmark-based ESWT based on anatomical palpation. Both groups underwent three sessions (2000 impulses at 2.2 bars, energy level 5, 8 Hz). Clinical outcomes were assessed using the Constant–Murley score (CMS) at baseline, 12 weeks, and 6 months. Calcific deposit resorption was evaluated via ultrasound imaging. **Results**: The ultrasound-guided group showed a significant improvement in CMS from a median of 50 (range: 30–75) at baseline to 97 (52–100) at 6 months. The landmark-based group also improved, from 48 (32–74) to 79 (40–96). At 6 months post-treatment, 90.9% of patients in the ultrasound-guided group achieved successful outcomes (CMS ≥ 86), compared to 50% in the landmark-based group. Complete calcific resorption occurred in 65.9% of patients in Group 1, compared to 50% in Group 2; 15% of patients in Group 2 showed no resorption. **Conclusions**: Ultrasound-guided ESWT was significantly more effective than landmark-based ESWT in improving shoulder function, reducing pain, and promoting calcific deposit resorption. These findings support ultrasound guidance as a preferred approach for optimizing ESWT outcomes in patients with calcific tendinopathy of the shoulder.

## 1. Introduction

Calcific tendinopathy of the rotator cuff is a frequent cause of chronic shoulder pain, often impairing quality of life and limiting functional mobility [1,2]. It is characterized by hydroxyapatite crystal deposition within the rotator cuff tendons, most commonly in the supraspinatus tendon [3,4]. While the exact cause remains unclear, contributing factors include chronic degeneration, metabolic dysfunction, and local tendon hypoxia [5,6].

Conservative treatments—such as nonsteroidal anti-inflammatory drugs (NSAIDs), physiotherapy, and subacromial corticosteroid injections—mainly offer symptomatic relief without resolving the underlying calcific deposits [7,8]. In refractory cases, surgical interventions like arthroscopic calcium removal or needling may be considered, though they are associated with longer recovery times and potential complications [9,10,11,12,13].

Extracorporeal shock wave therapy (ESWT) has gained attention as a non-invasive and effective alternative for treating calcific tendinopathy [14,15]. ESWT combines mechanical and biological effects that help reduce pain, promote tendon healing, and enhance calcification resorption [16,17]. It stimulates neovascularization through vascular endothelial growth factor expression, increases collagen (types I and III) synthesis, and modulates inflammatory pathways by reducing IL-6 and TNF-α levels [18,19,20,21,22,23]. In addition, cavitation effects from the shock waves promote fragmentation and clearance of calcific deposits [24,25].

Although ESWT is widely used, the optimal delivery technique remains under debate. Precise localization using ultrasound guidance allows shock waves to be focused directly on the calcific deposit, maximizing mechanical effects and stimulating localized biological responses. This focused energy delivery minimizes dispersion to adjacent healthy tissues, potentially enhancing efficacy and reducing side effects. Real-time imaging also enables dynamic adjustment of probe positioning, accommodating anatomical variability and calcific morphology—advantages not achievable with landmark-based approaches. This precision may improve calcification fragmentation, accelerate resorption, and support tendon healing [26,27]. In contrast, landmark-based techniques rely on surface anatomy, which may result in inconsistent energy delivery. However, few studies have directly compared the clinical effectiveness of these two methods [28,29].

This study addresses that gap by evaluating and comparing clinical and imaging outcomes between ultrasound-guided and landmark-based ESWT in patients with calcific shoulder tendinopathy. The goal is to determine whether image-guided targeting leads to superior pain relief, faster functional recovery, and more effective deposit resorption.

## 2. Materials and Methods

### 2.1. Study Design and Setting

This study was a prospective, controlled interventional clinical trial conducted at the Outpatient Physical Rehabilitation Center in Arad, Romania, between October 2023 and March 2025. The primary objective was to evaluate the clinical efficacy of ESWT in patients diagnosed with ultrasound-confirmed calcific tendinopathy of the shoulder.

Although randomization and allocation concealment were implemented, the study did not include a traditional control group (e.g., placebo or sham intervention). Instead, it compared two active treatment modalities—ultrasound-guided versus landmark-based ESWT. To reduce bias and confounding, sex-stratified randomization, a standardized treatment protocol, and blinded outcome assessment were employed. All procedures were performed by the same experienced clinician to ensure consistency.

The study was conducted in accordance with the Declaration of Helsinki and received approval from the institutional ethics committee (Approval No. 232/1/02 October 2023). While it was not registered in a formal clinical trial registry, the design adhered to the methodological standards of interventional clinical trials.

### 2.2. Study Population

A total of 134 patients were screened for eligibility. Fifty patients were excluded based on predefined criteria or declined participation. The final cohort consisted of 84 patients, who were enrolled and randomized into two treatment groups.

To ensure appropriate patient selection, strict inclusion and exclusion criteria were applied. Patients had to meet the specified clinical and imaging requirements for study enrollment, while conditions that could interfere with treatment efficacy were considered exclusion factors. A comprehensive list of these criteria is provided in Table 1. Contraindications to ESWT included active infection at the treatment site, coagulation disorders or current anticoagulant therapy, pregnancy, malignancy, the presence of a pacemaker, and severe cardiovascular or neurological disorders.

### 2.3. Clinical Evaluation

Demographic data, including age and gender, were collected for all 84 study participants.

Clinical assessment was performed using the Constant–Murley score (CMS), a validated tool for evaluating shoulder function, pain levels, movement, strength, and ability to perform daily activities [30,31]. CMS evaluations were conducted at baseline, 12 weeks, and 6 months post-treatment to assess functional outcomes.

A detailed breakdown of the CMS scoring components, total scoring criteria, and interpretation thresholds is provided in Supplementary File S1.

Although the original version of the CMS was used without modifications, the scale is widely accepted in European clinical settings and has been extensively applied in shoulder rehabilitation research. While formal validation studies for the Romanian population are limited, the CMS demonstrates strong face and content validity. Its consistent use by trained clinicians in this study ensured reliable application and interpretation of results.

To assess treatment efficacy, ultrasound imaging was performed at baseline, 12 weeks, and 6 months post-treatment to measure the size and location of calcific deposits. All evaluations were conducted by the same trained and blinded assessor to ensure measurement reliability.

### 2.4. Randomization and Intervention

Between October 2023 and March 2025, 84 patients (52 women and 32 men; mean age 56 years, range: 40–66 years) were enrolled and randomized into two intervention groups. A computer-generated randomization sequence, using permuted blocks of four and six, ensured balanced allocation. To account for the unequal gender distribution, randomization was stratified by sex, meaning that male and female participants were randomized separately to ensure proportional representation of each sex in both treatment groups. Allocation concealment was maintained using sealed, opaque, sequentially numbered envelopes, prepared by an independent researcher not involved in recruitment or treatment.

- Group 1 (*n* = 44): Received ultrasound-guided ESWT, with shock waves precisely targeted to the calcific deposit using real-time imaging.
- Group 2 (*n* = 40): Received landmark-based ESWT, applied at the presumed origin of the supraspinatus tendon based on surface anatomical landmarks.

**Figure 1:**
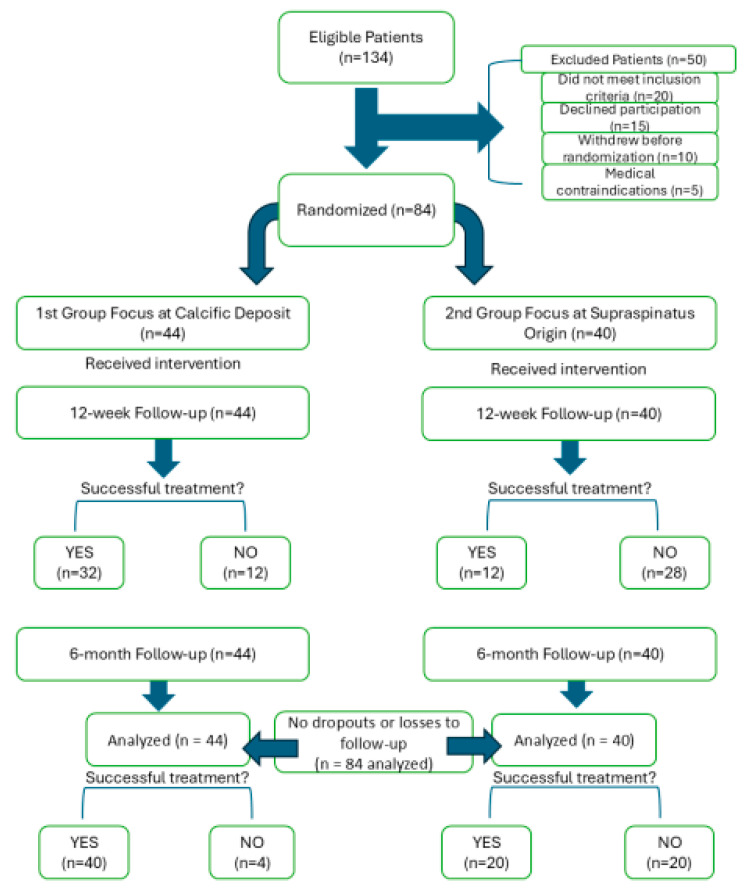
CONSORT flow diagram showing patient enrollment, randomization, intervention, follow-up, and treatment outcomes in the two study groups.

All participants completed their assigned treatments and follow-up. The CONSORT flow diagram shown in Figure 1 outlines the enrollment, allocation, intervention, and outcome assessment.

Due to the visible differences in procedures, patient blinding was not feasible. However, outcome assessors were blinded to group allocation. Additionally, both groups received identical treatment protocols (device type, dosage, frequency), administered by a qualified physiotherapist with substantial experience in musculoskeletal ultrasound and shock wave therapy, to minimize performance bias and inter-operator variability.

For Group 2, anatomical landmark-based ESWT was applied using surface palpation techniques to locate the supraspinatus tendon origin. The patient was positioned in a seated posture with the shoulder in hyperextension and internal rotation, a configuration supported by prior studies for enhancing access to the supraspinatus tendon and promoting calcific deposit resorption [1,32]. The acromion served as the primary bony reference point, and the application site was identified approximately 1–2 cm inferior and anterior to the anterolateral acromion, at the level of the greater tuberosity. Manual palpation for tenderness and tissue resistance, along with patient feedback, guided applicator placement.

**Figure 2:**
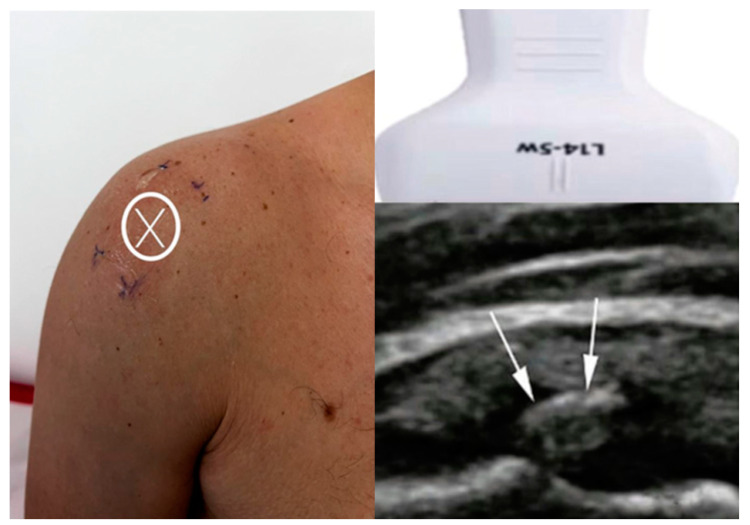
Ultrasound-guided targeting in Group 1. The X mark indicates the skin projection of the calcific deposit, and the arrows highlight the deposit on ultrasound imaging.

For Group 1, the arm position was dynamically adjusted based on real-time ultrasound visualization to optimally expose the calcific deposit, enabling accurate targeting regardless of its orientation or depth. This approach ensured maximal energy delivery directly to the lesion and enhanced treatment precision. As shown in Figure 2, the image on the left illustrates the X mark, which indicates the skin projection site of the calcific deposit, aligned with the center line of the ultrasound probe. The image on the bottom right shows the ultrasound scan, with white arrows highlighting the hyperechoic calcific deposit within the supraspinatus tendon.

All participants underwent three radial extracorporeal shock wave therapy (rESWT) sessions using an Intelect RPW Lite device (Chattanooga, DJO Global, Vista, CA, USA), administered one week apart, each delivering 2000 impulses at 2.2 bars, energy level 5, and 8 Hz frequency.

### 2.5. Ethical Considerations

This study was conducted in accordance with the International Conference on Harmonisation Good Clinical Practice guidelines and the Declaration of Helsinki. Ethical approval was obtained from the Institutional Review Board of Davima Clinic, Arad, Romania (Approval No. 232/1/02 October 2023). Although not formally registered in a clinical trials registry, the protocol and methods adhered to international standards for interventional studies.

Before enrollment, all patients provided written informed consent after receiving a detailed explanation of the study objectives, procedures, potential risks, and benefits. Patient confidentiality and data protection were ensured in compliance with applicable regulations, and all personal health information was anonymized and securely stored.

### 2.6. Statistical Analysis

Before choosing appropriate statistical tests, data normality was assessed using the Kolmogorov–Smirnov test, and homogeneity of variances was examined to determine the suitability of parametric analyses.

A sample size calculation was performed using G*Power software (version 3.1.9.7), assuming a moderate effect size (Cohen’s d = 0.65), an alpha level of 0.05, and a power of 0.80. Based on this, a minimum of 38 participants per group was required. To account for potential dropouts, a total of 84 patients were enrolled.

Statistical analyses were conducted using SPSS version 10.0 (SPSS Inc., Chicago, IL, USA). Continuous data are presented as mean ± standard deviation. Repeated measures ANOVA was used to assess changes in the CMS over time (baseline, 12 weeks, and 6 months) within each group, provided data met normality assumptions. Between-group comparisons at each time point were performed using Bonferroni-adjusted post hoc tests. For non-normally distributed variables, the Friedman test was employed as a non-parametric alternative.

To control for Type I error across multiple comparisons, Bonferroni correction was applied, setting the adjusted significance threshold at *p* < 0.017. Only results meeting this criterion are reported as statistically significant.

Cohen’s d was used to calculate effect sizes, interpreted as small (0.2), moderate (0.5), or large (≥0.8).

Additionally, binary logistic regression analysis was conducted to identify predictors of treatment success, defined as achieving a CMS ≥ 86 at six months. Independent variables included treatment group, age, sex, baseline CMS, and initial calcific deposit size. Odds ratios with 95% confidence intervals and *p*-values are reported. A significance level of *p* < 0.05 was used for regression analyses.

All analyses followed a per-protocol approach, as no dropouts or protocol deviations occurred during the study.

### 2.7. Follow-Up Protocol and Study Endpoints

All patients followed a structured follow-up protocol to assess treatment efficacy and safety. Clinical and imaging evaluations were performed at baseline, 12 weeks, and 6 months post-treatment. All assessments were conducted by the same blinded assessor to reduce potential bias.

Ultrasound imaging was used to evaluate calcific deposit size and location, while functional assessments were performed using the CMS scoring system. Patients were monitored for potential adverse events related to ESWT treatment.

The primary study endpoint was pain reduction and functional improvement, as measured by CMS scores at 6 months post-treatment. The secondary endpoints included the following:

- Calcific deposit resorption rate, assessed via ultrasound imaging.
- Symptom recurrence at the 6-month follow-up.

### 2.8. Hypotheses of the Study

This study aimed to compare the clinical efficacy and imaging outcomes of ultrasound-guided versus landmark-based ESWT in the treatment of calcific tendinopathy of the shoulder.

- Primary hypothesis: There is a statistically significant difference in pain relief and functional improvement between ultrasound-guided and landmark-based ESWT, as measured by the CMS at 6 months post-treatment. This hypothesis is supported by prior studies suggesting that ultrasound-guided ESWT may allow more accurate targeting of calcific deposits, potentially improving treatment outcomes [32,33,34].
- Secondary hypothesis: There is a statistically significant difference in the rate of calcific deposit resorption between the two ESWT methods, as assessed by ultrasound imaging. Previous studies have demonstrated improved resorption with image-guided techniques, although direct comparisons between methods remain limited [28,35].

## 3. Results

### 3.1. Clinical Outcome Measures

Given that outcomes were assessed at multiple time points within the same individuals, repeated measures ANOVA was employed to account for within-subject variability and test for interaction effects between time and treatment group.

Functional improvement and pain reduction were observed in both treatment groups over time; however, Group 1 (ultrasound-guided ESWT) demonstrated statistically and clinically superior outcomes compared to Group 2 (landmark-based ESWT).

Repeated measures ANOVA revealed a significant interaction between time and group (*p* < 0.001), indicating that the rate of improvement in CMS differed significantly between the two groups across the three time points (baseline, 12 weeks, and 6 months). Post hoc comparisons with Bonferroni adjustment showed that at both 12 weeks and 6 months, Group 1 exhibited significantly higher CMS scores compared to Group 2 (*p* < 0.017). No significant difference was observed at baseline (*p* > 0.017).

At 12 weeks, the median CMS score was 94.5 (range: 45–100) in Group 1 and 70 (35–98) in Group 2. At 6 months, these scores further improved to 97 (52–100) in Group 1 and 79 (40–96) in Group 2. The between-group effect sizes were moderate at 12 weeks (Cohen’s d = 0.75) and large at 6 months (Cohen’s d = 1.18), underscoring the clinical relevance of these findings.

Treatment success rates and subjective improvement also favored ultrasound-guided ESWT. At 6 months, the success rate was significantly higher in Group 1 (64.2% ± 27.1) compared to Group 2 (35.8% ± 29.4), with a 95% confidence interval of 12.3 to 42.1 (*p* < 0.017, Bonferroni-adjusted). Subjective improvement mirrored this pattern, with 90.9% ± 9.6 of patients in Group 1 reporting clinical benefit, compared to 50.0% ± 20.0 in Group 2 (CI: 28.5 to 47.8; *p* < 0.017, Bonferroni-adjusted). A detailed summary of all clinical outcome measures is presented in Table 2.

These results confirm the clinical efficacy of ultrasound-guided ESWT, demonstrating significantly greater improvements in both objective functional outcomes and subjective patient-reported measures compared to the landmark-based approach.

### 3.2. Comparison of Functional and Pain Improvement Between Groups

Although both groups exhibited pain relief and functional improvement, the ultrasound-guided ESWT group (Group 1) demonstrated statistically superior outcomes at the 6-month follow-up.

At 6 months, the majority of the patients (90.90%) in the ultrasound-guided ESWT group (Group 1, *n* = 44) achieved successful treatment outcomes, with 54.6% reaching excellent functional recovery (CMS ≥ 86). In contrast, 50% of patients in the landmark-based ESWT group (Group 2, *n* = 40) remained in the “fair” category, and 50% still experienced poor outcomes.

A chi-square test of independence revealed a statistically significant association between treatment group and CMS category at 6 months (χ^2^ = 36.21, *p* < 0.001), indicating that ultrasound-guided ESWT was significantly more likely to result in favorable functional outcomes.

These findings indicate that ultrasound-guided ESWT provides superior clinical benefits, including greater pain relief, enhanced functional recovery, and improved patient-reported outcomes in calcific tendinopathy of the shoulder. The overall CMS outcomes at 6 months post-treatment are summarized in Table 3.

These results confirm that ultrasound-guided ESWT leads to significantly better functional recovery and pain reduction compared to landmark-based ESWT, particularly at the 6-month follow-up. The distribution of CMS categories illustrates a clear advantage of ultrasound-guided ESWT, with over half of the patients achieving excellent functional outcomes.

Furthermore, no patients in the landmark-based group attained excellent outcomes, emphasizing the clinical superiority of ultrasound-guided ESWT for calcific tendinopathy treatment.

The between-group effect size for CMS improvements was moderate at 12 weeks (Cohen’s d = 0.75) and large at 6 months (Cohen’s d = 1.18), indicating a meaningful clinical benefit of ultrasound-guided ESWT over the landmark-based approach. The median CMS scores also reflected these differences, as shown in Table 4.

### 3.3. Calcium Deposit Resorption Analysis

In this study, partial resorption was defined based on characteristic ultrasound findings, including a reduction in calcification size accompanied by visible fragmentation, irregular or blurred contours, and heterogeneous echogenicity within the deposit. Posterior acoustic shadowing was typically reduced or irregular, suggesting a decrease in density and softening of the calcific material. These changes indicated an ongoing breakdown of the deposit, without its complete disappearance.

Calcium deposit resorption was significantly higher in Group 1 (ultrasound-guided ESWT) compared to Group 2 (landmark-based ESWT). At the 6-month follow-up, a greater proportion of patients in Group 1 exhibited complete or partial resorption, while half of the patients in Group 2 showed no resorption (Table 5).

- Complete resorption occurred in 65.9% (29/44) of patients in Group 1, compared to 50% (20/40) in Group 2.
- Partial resorption was observed in 34.1% (15/44) of Group 1 and 35% (14/40) of Group 2 patients.
- No resorption was seen in 0% of patients in Group 1, whereas 15% (6/40) of Group 2 patients showed no change in calcific deposits.

A chi-square test of independence confirmed a significant association between treatment group and resorption category at 6 months (χ^2^ = 9.52, *p* = 0.009), indicating a higher likelihood of favorable resorption outcomes in the ultrasound-guided group.

These findings suggest that ultrasound-guided ESWT significantly improves calcific deposit resorption, whereas landmark-based ESWT results in lower resolution rates. The ability to precisely target the calcified area using ultrasound guidance likely enhances mechanical fragmentation and biological resorption.

### 3.4. Calcific Deposit Size and Location—Imaging Results

Ultrasound imaging revealed a significant reduction in the size of calcific deposits over time in both treatment groups, with a more pronounced reduction observed in the ultrasound-guided ESWT group (Group 1). At baseline, the median calcific deposit size was 12.5 mm (range: 8.3–18.4 mm) in Group 1 and 12.1 mm (range: 7.9–17.2 mm) in Group 2. By 6 months, the median size decreased to 2.0 mm (range: 0–6.2 mm) in Group 1 and 5.1 mm (range: 1.8–10.4 mm) in Group 2.

A repeated measures ANOVA revealed a statistically significant interaction between time and group (*p* < 0.001), indicating that the reduction in deposit size over time differed significantly between the two groups. Post hoc comparisons with Bonferroni adjustment showed significant differences between groups at both 12 weeks and 6 months (*p* < 0.017), while no significant difference was observed at baseline (*p* > 0.017) (Table 6).

These findings underscore the greater efficacy of ultrasound-guided ESWT in promoting the resorption and reduction of calcific deposits compared to landmark-based treatment.

**Figure 3:**
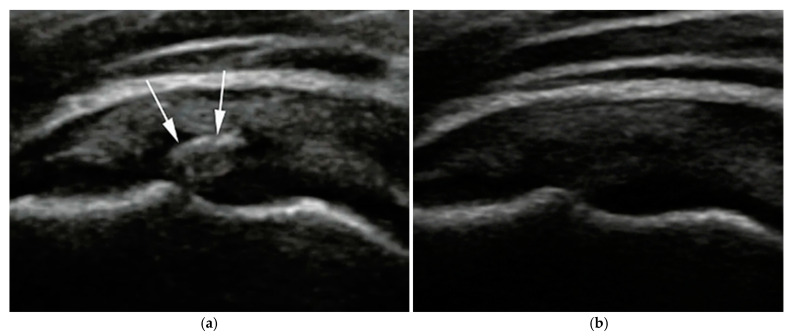
Representative ultrasound images of calcific deposits in a patient treated with ultrasound-guided ESWT. (**a**) Baseline image showing a well-defined calcific deposit (white arrows); (**b**) Six-month follow-up demonstrating complete resorption, with no visible echogenic material.

Calcific deposits were most commonly located at the supraspinatus tendon insertion. Follow-up imaging in the ultrasound-guided group revealed echotexture changes and fragmentation consistent with progressive resorption (Figure 3).

Both ultrasound-guided and landmark-based ESWT were generally well tolerated. No serious adverse events were observed. Minor, transient side effects such as localized pain or mild bruising were occasionally reported, consistent with the known safety profile of ESWT.

### 3.5. Predictors of Successful Treatment Outcome

A binary logistic regression was performed to assess the impact of various factors on the likelihood of achieving a successful treatment outcome (CMS ≥ 86 at 6 months). The model included treatment group, age, sex, baseline CMS, and initial calcific deposit size as predictors.

The overall model was statistically significant (χ^2^ = 29.6, df = 5, *p* < 0.001), correctly classifying 81.2% of cases. Treatment group emerged as the strongest independent predictor. Patients treated with ultrasound-guided ESWT were significantly more likely to achieve success compared to those in the landmark-based group (OR = 5.42; 95% CI: 2.13–13.76; *p* < 0.001). Baseline CMS was also significant (OR = 1.09 per point increase; 95% CI: 1.01–1.17; *p* = 0.03), while age, sex, and calcific deposit size were not. The results of the logistic regression analysis are summarized in Table 7.

## 4. Discussion

This interventional clinical trial provides compelling evidence that ultrasound-guided ESWT leads to superior outcomes compared to landmark-based ESWT in the treatment of calcific tendinopathy of the shoulder. Patients receiving ultrasound-guided treatment experienced significantly greater improvements in pain relief, functional recovery, and calcific deposit resorption. These benefits are primarily attributed to the real-time visualization and precision targeting of the shock wave energy, which enables accurate alignment with the pathological calcific focus and minimizes unnecessary exposure to adjacent healthy tissue [36,37].

This targeted delivery enhances the mechanical fragmentation of calcific deposits and promotes a more robust biological response, including localized neovascularization, tenocyte stimulation, collagen type I and III synthesis, and modulation of pro-inflammatory cytokines such as IL-6 and TNF-α. In contrast, the landmark-based approach lacks this precision, resulting in inconsistent energy distribution and suboptimal activation of these therapeutic mechanisms. The dynamic feedback loop provided by ultrasound imaging allows for adaptive probe positioning, accounting for variations in tendon anatomy, deposit location, and patient-specific characteristics.

These results support the growing recognition that image-guided techniques, particularly in soft-tissue disorders, can optimize treatment efficacy by aligning the physical effects of therapy with specific tissue targets. The findings also contribute to the expanding evidence base supporting ultrasound-guided interventions as a standard of care in musculoskeletal rehabilitation [38,39].

### 4.1. Clinical and Functional Outcomes

Patients who received ultrasound-guided ESWT demonstrated significantly higher CMSs at both 12 weeks and 6 months. This improvement is likely attributable to enhanced biological responses, including neovascularization, modulation of inflammation, and stimulation of tendon healing, which occur when energy is delivered precisely to the affected tissue [40,41,42,43].

The success rate in the ultrasound-guided group was 90.9%, compared to 50% in the landmark-based group, reinforcing the importance of accurate targeting in ESWT. These results are in line with prior research demonstrating the clinical advantages of imaging-guided approaches over palpation-based techniques [44,45,46,47].

Beyond statistical significance, the improvements observed in the CMS scores have clear clinical importance. Patients in the ultrasound-guided group improved by more than 45 points over 6 months—well above the minimal clinically important difference (MCID), typically reported at 10–15 points. These gains reflect substantial, real-world improvements in shoulder function and quality of life. Additionally, the large effect size observed at 6 months (Cohen’s d = 1.18) further supports the meaningful impact of ultrasound-guided ESWT.

### 4.2. Calcific Deposit Resorption

Ultrasound-guided ESWT also led to significantly greater calcific deposit resorption at 6 months. None of the patients in this group had persistent calcifications, while 50% of the landmark-based group showed no significant resorption. These findings highlight the importance of accurate energy delivery for effective mechanical disruption and resorption of calcific deposits [48,49,50].

Furthermore, procedures such as ultrasound-guided barbotage and needling have shown similar success, supporting the broader value of image-guided techniques in managing calcific tendinopathy [51,52].

### 4.3. Clinical Implications and Future Directions

This study highlights the clinical value of ultrasound-guided ESWT as a non-invasive and effective treatment for calcific tendinopathy of the shoulder. By enabling precise localization of calcific deposits under real-time imaging, ultrasound guidance enhances mechanical energy delivery, leading to superior pain relief, faster functional recovery, and more consistent calcification resorption compared to landmark-based approaches [26,53].

Ultrasound-guided ESWT minimizes energy dispersion and procedural variability by allowing accurate, dynamic adjustment of the applicator during treatment. This precision not only improves fragmentation of calcific material but also enhances key biological responses such as neovascularization, inflammatory modulation, and tendon remodeling [54,55,56]. The high treatment success rate observed in the ultrasound-guided group (90.9%) underscores its clinical applicability.

Given its favorable outcomes and reproducibility, ultrasound-guided ESWT should be considered a second-line intervention, particularly for patients who do not respond to NSAIDs or physiotherapy. Early implementation after the failure of conservative measures may help prevent chronic symptom progression and reduce the need for surgical intervention. Longitudinal monitoring using validated tools such as the CMS and ultrasound imaging is recommended to assess recovery and detect recurrence.

Individual variability in treatment response should also be acknowledged. Psychological and behavioral factors—including anxiety, pain sensitivity, and physical activity—may influence recovery trajectories. A multidisciplinary approach that integrates physiotherapy, patient education, and psychosocial support could enhance treatment success [57,58,59].

Future research should focus on large-scale interventional clinical trials with extended follow-up periods, including head-to-head comparisons with corticosteroid injections, physical therapy, or a placebo. Investigating optimal treatment parameters (e.g., energy levels, frequency, session count) and identifying patient subgroups most likely to benefit based on calcification characteristics or comorbidities will support personalized care.

Finally, evaluating the cost-effectiveness of ultrasound-guided ESWT and exploring the use of emerging technologies—such as biomechanical imaging and AI-guided probe positioning—may further improve precision, accessibility, and treatment efficiency.

### 4.4. Study Limitations

Despite the encouraging outcomes, several limitations of this study should be acknowledged.

First, the absence of a control group limits the ability to compare ultrasound-guided ESWT with alternative interventions, such as placebo, corticosteroid injections, or structured physiotherapy programs. Without a non-treatment or placebo comparator, it is difficult to isolate the true therapeutic effect of ESWT from placebo responses or the natural course of the condition. Future randomized controlled trials should include a control arm to strengthen causal inferences.

Second, the relatively small sample size (*n* = 84) may restrict the statistical power and generalizability of the findings. Although significant differences were observed between the groups, larger multicenter trials are necessary to validate these results across more diverse patient populations and clinical settings.

Third, the follow-up period was limited to six months. While meaningful improvements in clinical and imaging outcomes were observed during this time, longer follow-up is needed to assess the durability of treatment effects, recurrence rates, and potential long-term adverse events. Future studies should consider follow-up durations of at least 12 months.

Lastly, psychosocial factors—including anxiety, depression, and fear-avoidance beliefs—were not evaluated. These variables can influence pain perception, functional outcomes, and patient adherence to rehabilitation. Integrating standardized psychosocial assessments in future studies would allow for a more holistic understanding of patient recovery and could inform multidisciplinary treatment approaches.

## 5. Conclusions

This study demonstrates that ultrasound-guided ESWT is more effective than landmark-based ESWT in treating calcific tendinopathy of the shoulder. The precise localization achieved through ultrasound guidance results in greater pain relief, improved functional recovery, and enhanced calcific deposit resorption compared to non-guided application.

These findings support the primary hypothesis that ultrasound-guided ESWT leads to significantly better clinical outcomes, as reflected in higher CMSs at 6 months post-treatment. The secondary hypothesis is also supported, as ultrasound guidance facilitated greater resorption of calcific deposits, confirmed via imaging. Therefore, the null hypothesis is rejected.

Clinically, these results suggest that ultrasound-guided ESWT should be considered the preferred non-invasive intervention for symptomatic calcific tendinopathy, particularly in patients who have not responded to conservative therapies. Integration of ultrasound imaging into ESWT procedures enhances treatment precision and effectiveness, potentially reducing the risk of symptom recurrence and improving patient quality of life.

However, this study has limitations, including the absence of a control group, a relatively small sample size, and limited follow-up duration. Future research should include large-scale, randomized controlled trials with control arms and extended follow-up periods to confirm long-term benefits. Additionally, incorporating multimodal rehabilitation strategies—including physiotherapy, patient education, and psychological support—may further improve outcomes and prevent chronic disability.

In summary, ultrasound-guided ESWT is a safe, effective, and clinically superior approach for managing calcific tendinopathy of the shoulder. Standardizing ultrasound-guided ESWT protocols in clinical practice could improve treatment consistency and optimize outcomes for affected patients.

## Figures and Tables

**Table 1:** Inclusion and exclusion criteria.

Inclusion Criteria	Exclusion Criteria
Adults aged ≥ 18 years	Patients diagnosed with rotator cuff tears
Diagnosed calcific tendinopathy, confirmed via ultrasound imaging	Patients diagnosed with glenohumeral arthritis or instability
Preserved shoulder range of motion, defined as ≥90° abduction with unrestricted rotation	Patients diagnosed with neurological disorders affecting the shoulder
Persistent symptoms for at least six months despite conservative management (NSAIDs, physiotherapy, subacromial injections)	Pregnancy, contraindications to ESWT, previous ESWT, or prior surgical intervention
Patients who voluntarily provided informed consent for participation in the study	Patients who declined to provide informed consent for participation in the study

NSAIDs—nonsteroidal anti-inflammatory drugs; ESWT—extracorporeal shock wave therapy.

**Table 2:** Comparison of CMS, treatment success, and subjective improvement between study groups.

Group/Parameter	Group 1 (Ultrasound-Guided ESWT, *n* = 44)	Group 2 (Landmark-Based ESWT, *n* = 40)	Between-Group Median Difference (95% CI)	Statistical Significance
CMS—median (range)				
Before intervention	50 (30–75)	48 (32–74)	2.0 (–3.5 to 8.2)	*p* > 0.017
12 weeks	94.5 (45–100)	70 (35–98)	24.5 (10.6 to 31.5)	*p* < 0.017
6 months	97 (52–100)	79 (40–96)	18.0 (11.8 to 27.9)	*p* < 0.017
Number of successful treatments (% mean ± SD)				
12 weeks	95.1 ± 16.3	78.5 ± 20.1	11.8 to 27.9	*p* < 0.017
6 months	64.2 ± 27.1	35.8 ± 29.4	12.3 to 42.1	*p* < 0.017
Subjective improvement (% mean ± SD)				
12 weeks	64.2 ± 27.1	35.8 ± 29.4	12.3 to 42.1	*p* < 0.017
6 months	90.9 ± 9.6	50.0 ± 20.0	28.5 to 47.8	*p* < 0.017

ESWT–extracorporeal shock wave therapy; CMS—Constant–Murley score. Footnote: Bonferroni-adjusted significance threshold. The 95% CI reflects the between-group difference in median CMS.

**Table 3:** Overall CMS outcomes at 6 months post-ESWT.

CMS Outcome	Group 1 (Ultrasound-Guided ESWT, *n* = 44)	Group 2 (Landmark-Based ESWT, *n* = 40)
Excellent (86–100 CMS points)	54.6% (24/44)	0% (0/40)
Good (71–85 CMS points)	27.5% (12/44)	10% (4/40)
Fair (56–70 CMS points)	8.9% (4/44)	70% (16/40)
Poor (0–55 CMS points)	8.9% (4/44)	20% (20/40)
Chi-square (χ^2^) test result	χ^2^ = 36.21, *p* < 0.001	

ESWT—extracorporeal shock wave therapy; CMS–Constant–Murley score.

**Table 4:** CMS and effect size at 12 weeks and 6 months post-treatment.

Time Point	Group 1 (Ultrasound-Guided ESWT, *n* = 44)	Group 2 (Landmark-Based ESWT, *n* = 40)	*p*-Value	Effect Size (Cohen’s d)
12 weeks post-treatment	94.5 (45–100)	70 (35–98)	<0.017	0.75
6 months post-treatment	97 (52–100)	79 (40–96)	<0.017	1.18

ESWT—extracorporeal shock wave therapy. Footnote: Bonferroni-adjusted significance threshold applied; effect sizes based on group mean differences.

**Table 5:** Calcific deposit resorption at 6 months post-ESWT.

Resorption Category	Group 1 (Ultrasound-Guided ESWT, *n* = 44)	Group 2 (Landmark-Based ESWT, *n* = 40)
Complete resorption	29 (65.9%)	20 (50.0%)
Partial resorption	15 (34.1%)	14 (35.0%)
No resorption	0 (0%)	6 (15.0%)
Chi-square (χ^2^) test	χ^2^ = 9.52, *p* = 0.009	

ESWT—extracorporeal shock wave therapy.

**Table 6:** Mean calcific deposit size at baseline, and 12 weeks and 6 months post-rreatment.

Time Point	Group 1—Ultrasound-Guided ESWT	Group 2—Landmark-Based ESWT	*p*-Value
Baseline	12.5 (8.3–18.4)	12.1 (7.9–17.2)	>0.017
12 weeks post-treatment	5.1 (2.4–10.7)	7.8 (3.1–12.3)	<0.017
6 months post-treatment	2.0 (0–6.2)	5.1 (1.8–10.4)	<0.017

ESWT—extracorporeal shock wave therapy. Statistical analysis: Repeated measures ANOVA used to assess group × time interaction; Bonferroni correction applied for post hoc comparisons (adjusted significance threshold *p* < 0.017).

**Table 7:** Logistic regression analysis of predictors of treatment success (CMS ≥ 86 at 6 months).

Predictor	Odds Ratio	95% Confidence Interval	*p*-Value
Treatment group (ultrasound vs. landmark)	5.42	2.13–13.76	<0.001
Age (years)	0.98	0.93–1.03	0.38
Sex (female = 1)	1.21	0.52–2.81	0.65
Baseline CMS	1.09	1.01–1.17	0.03
Calcific deposit size (mm)	0.94	0.81–1.09	0.42

## Data Availability

The raw data supporting the conclusions of this article will be made available by the authors on request.

## Supplementary Materials

The following supporting information can be downloaded at: https://www.mdpi.com/article/10.3390/diagnostics15091142/s1, Supplementary File S1. Detailed Description of the Constant-Murley Score (CMS). References [60,61,62] are cited in the Supplementary Materials.

## Abbreviations

The following abbreviations are used in this manuscript:

NSAIDs	Nonsteroidal anti-inflammatory drugs
ESWT	Extracorporeal shock wave therapy
CMS	Constant–Murley score
rESWT	Radial extracorporeal shock wave therapy
MCID	Minimal clinically important difference
