# Supplementary material for: Ultrasound-Guided Versus Landmark-Based Extracorporeal Shock Wave Therapy for Calcific Shoulder Tendinopathy: An Interventional Clinical Trial
Source: Diagnostics (Basel). 2025 Apr 30;15(9):1142. doi: 10.3390/diagnostics15091142 (PMC12071427; doi:10.3390/diagnostics15091142)
Supplement: Supplementary file 1 [file diagnostics-15-01142-s001.zip › diagnostics-3583507-supplementary.pdf]

# Supplementary File S1. Detailed Description of the Constant-Murley Score (CMS)

The Constant-Murley Score (CMS) is a widely used, validated tool for evaluating shoulder function in clinical and research settings. It assesses pain, daily activities, strength, and range of motion:

- **Pain:** up to 15 points
- **Activities of Daily Living (ADL):** up to 20 points
- **Strength:** up to 25 points
- **Range of Motion (ROM):** up to 40 points
  *(ROM includes forward elevation, external rotation, abduction, and internal rotation)*

**Maximum Total Score:** 100 points
A higher score indicates better shoulder function.

**Interpretation of CMS values:**
- **0–55 points:** Poor
- **56–70 points:** Fair
- **71–85 points:** Good
- **86–100 points:** Excellent

**Note:** This scoring system was used at baseline, 12 weeks, and 6 months to monitor functional improvement after treatment.

**Reference:**


1. Bejer, A.; Płocki, J.; Probachta, M.; Kotela, I.; Kotela, A. A Comparison Study of the Western Ontario Rotator Cuff Index, and the Constant–Murley Score with Objective Assessment of External Rotator Muscle Strength and Pain in Patients after Arthroscopic Rotator Cuff Repair. *Int. J. Environ. Res. Public Health* **2023**, *20*, 6316. https://doi.org/10.3390/ijerph20136316.
2. Jo, Y.H.; Lee, K.H.; Jeong, S.Y.; Kim, S.J.; Lee, B.G. Shoulder outcome scoring systems have substantial ceiling effects 2 years after arthroscopic rotator cuff repair. *Knee Surg Sports Traumatol Arthrosc* **2021**, *29*, 2070–2076. https://doi.org/10.1007/s00167-020-06036-y.
3. Louwerens, J.K.G.; van den Bekerom, M.P.J.; van Royen, B.J.; Eygendaal, D.; van Noort, A.; Sierevelt, I.N. Quantifying the minimal and substantial clinical benefit of the Constant-Murley score and the Disabilities of the Arm, Shoulder and Hand score in patients with calcific tendinitis of the rotator cuff. *JSES Int* **2020**, *4*, 606–611. https://doi.org/10.1016/j.jseint.2020.05.001.
